# FOXP Transcription Factors in Thyroid Cancer: From Molecular Expression to Clinical Significance

**DOI:** 10.3390/biomedicines14061222

**Published:** 2026-05-28

**Authors:** Tijana Vasiljević, Nikola Stevan Kokanov, Bojana Kožik

**Affiliations:** 1 Faculty of Medicine, University of Novi Sad, 21000 Novi Sad, Serbia; tijana.vasiljevic@mf.uns.ac.rs; 2 Department of Pathology and Laboratory Diagnostic, Oncology Institute of Vojvodina, 21204 Sremska Kamenica, Serbia; 3 Laboratory for Radiobiology and Molecular Genetics, Vinca Institute of Nuclear Sciences, National Institute of Republic of Serbia, University of Belgrade, 11000 Belgrade, Serbia; nikola.kokanov@vin.bg.ac.rs

**Keywords:** biomarkers, FOXP, prognosis, thyroid cancer, transcription factors

## Abstract

Thyroid cancer (TC) is the most common endocrine malignancy, with a steadily rising global incidence. Despite most cases having a favorable prognosis, a subset of patients develops aggressive, recurrent, or radioiodine-refractory disease, demonstrating the need for improved molecular biomarkers and targeted therapies. The Forkhead box P (FOXP) transcription factors (FOXP1–FOXP4) have appeared as important regulators of tumor biology, yet their roles in thyroid cancer remain incompletely defined. This review summarizes current bioinformatic, experimental, and clinical evidence regarding FOXP expression patterns, molecular mechanisms, and clinical relevance in TC. FOXP3 and FOXP4 are mainly associated with aggressive clinicopathological features, including extrathyroidal invasion, lymph node metastasis, and distant metastases, and may serve as markers of poor prognosis. The most explored FOXP3 contributes to immune evasion and radioiodine resistance by suppressing sodium iodide symporter expression and regulating tumor-associated immune responses. FOXP4 promotes tumor progression by activating key oncogenic signaling pathways and regulating non-coding RNAs. In contrast, evidence indicates that FOXP2 primarily acts as a tumor suppressor in TC by inhibiting cell proliferation and promoting apoptosis, although it may show context-dependent functions. FOXP1, though less well studied, is also suggested to have tumor-suppressive effects in some studies, and demands additional investigation in TC. Collectively, current evidence suggests that FOXP family members may represent promising diagnostic, prognostic, and therapeutic biomarkers in thyroid cancer, although further validation in large clinical cohorts and mechanistic studies is still required.

## 1. Introduction

Thyroid cancer (TC) remains the most common endocrine malignancy worldwide, ranking seventh in general population incidence, steadily increasing in time, according to the 2022 Global Cancer Statistics published by the International Agency for Research on Cancer. High female predominance with approximately three times higher female to male ratio places TC third most common malignancy in the female population, and only thirteenth in males. Although mortality rates have remained low and stable for decades, a worrisome increase inincidence among the middle-aged population (between 45 and 64 years) has been spotted [1]. Exposure to radiation, heavy metals, and air pollution hasbeen considered as predisposing factors for the constant growth in incidence, as well as modifiable risk factors such as obesity, lack of exercise, heavy alcohol consumption, smoking and secondhand smoking [2,3,4]. Simultaneous technological development with high-resolution ultrasound and structured guidelines in Thyroid Imaging Reporting and Data System (TI-RADS) of thyroid nodes, as well as personal interest in patients, have led to overdiagnosis of small thyroid cancers (<1 cm) in recent years [4,5].

The fifth edition of the World Health Organization (WHO) classification of endocrine tumors, released in 2022, gives a revised classification of thyroid neoplasms, integrating classic histology, immunohistochemical markers and molecular pathways into risk-stratified groups that influence clinical treatment [6,7]. Follicular cell-derived differentiated thyroid carcinomas (DTC) comprise the largest group of malignant neoplasms of the thyroid gland with distinct features of papillary thyroid carcinoma (PTC), follicular thyroid carcinoma (FTC) and oncocytic thyroid carcinoma (OTC). WHO classification gives two distinct high-grade follicular cell-derived carcinomas: poorly differentiated thyroid carcinoma (PDTC) and differentiated high-grade thyroid carcinoma (DHGTC), representing the intermediate stage between indolent DTC and the aggressive form of undifferentiated TC called anaplastic carcinoma (ATC).A frequent parafollicular cell-derived neoplasm is medullary thyroid carcinoma (MTC) [7].

Differentiated thyroid carcinoma is generally considered an indolent malignant neoplasm with excellent prognosis in adults. In children and young adults, aggressive behavior is reflectedin local metastases and high recurrence rates. However, mortality remains low even in this population [1,3,7]. Molecular pathway of PTC is complex and includes the MAPK signal pathway, inducing point mutations and activating BRAF-V600E mutation present in >60% classic subtype PTC [3,4,7]. Although they have a high affinity to lymph nodes, clinical outcome is dependent on the size of the tumor, leaving tumors smaller than 1 cm often clinically silent. Capsular or/and vascular invasion makes a distinction between benign follicular adenoma with excellent prognosis and FTC with risk-stratified groups, with vascular invasion giving the worst prognosis. FTC exhibits enhanced lipid metabolism and PI3K/AKT pathway activation and is usually a RAS-mutation-induced neoplasm, with NRAS being the most frequent driver mutation [4,7]. Unique histologic feature of OC is abundant oxyphilic cytoplasm, but the tumor generally shows similar features toFTC. These tumors show more local recurrence and distant metastases than classic FTC and are radioiodine refractory. The MAPK signal pathway with RAS mutation and TERT promoter mutations is the molecular basis of the tumor [3,7]. Medullary TC (<5%) is a neuroendocrine neoplasm with a characteristic RET proto-oncogene mutation [4,7]. Anaplastic carcinoma is a rare neoplasm (<2%) characterized by explosive and locally aggressive tumor growth as well as refractoriness to standard treatment, which leads to a very poor prognosis [7,8]. ATC is generally MAPK pathway driven, but also PI3K/AKT pathway induced, harboring more than one driver mutation.

Most of the TCs present asymptomatically, found incidentally on imaging. Nonetheless, fatigue, emotional distress, anxiety or depression, followed by hoarseness, compression or a noticeable lump in the neck can be present [3,4]. Surgery remains the gold standard of care [3,4,8], followed by radioactive iodine (RAI), and rarely chemotherapy and radiotherapy for radioiodine-refractory TCs [3,5]. Chemotherapy is rarely used in aggressive neoplasms such as ATC [5,8]. However, radioiodine-refractory carcinomas and recurrent metastases pose a great challenge for treatment, leading to exploring novel treatment options based on the molecular pathways and innovative treatments. Immunotherapies like PD-1 inhibitors enhance patients’immune systems. Tyrosine kinase inhibitors (TKIs) such as sorafenib, lenvatinib and vandetanib have been approved for treating aggressive TC. BRAFV600E inhibitors and their combination with MEK inhibitors can be effective for patients with BRAFV600E mutation in refractory PTC [5,8,9]. Treatments for specific mutations, such as TRK kinase inhibitors (larotrectinib and entrectinib), have proven to be successful in treating cancers with NTRK gene mutations. Pembrolizumab is a treatment option for tumors withmicrosatellite instability–high status or mismatch repair deficiencies [5,8,9,10].

Despite generally favorable outcomes, several clinical challenges persist in thyroid cancer, including overdiagnosis of indolent tumors, difficulties in distinguishing aggressive from low-risk disease, and the management of radioiodine-refractory and recurrent cases. Increasing evidence suggests that transcriptional and signaling regulators involved in tumor microenvironment modulation, oncogenic pathway activation, and treatment resistance may represent promising targets for precision oncology approaches in solid tumors [11,12]. In this context, FOXP family members have emerged as potential regulators of several biological processes highly relevant to thyroid cancer progression, including immune evasion, epithelial–mesenchymal transition, and dysregulated signaling pathway activity.

### 1.1. Structure and Function of FOXP Genes

The *FOXP* subfamily belongs to the larger Forkhead (*FOX*) gene superfamily and comprises four members: *FOXP1*(3p14.1), *FOXP2*(7q31), *FOXP3*(Xp11.23), and *FOXP4*(6p21.1) [13]. *FOX* genes are highly conserved across eukaryotes, occurring in organisms ranging from yeast to humans, with 43 *FOX* genes identified in the human genome. Many of these genes, including all members of the *FOXP* subfamily, play essential roles in development and organogenesis [14]. Deregulation of *FOX* genes hasbeen linked to cancer initiation, invasion, progression, and therapy resistance, as well as to the modulation of other oncogenic pathways that contribute to tumor development [15]. In particular, FOXP proteins are crucial for immune regulation, organ development, and cancer pathogenesis [14,16].

**Figure 1:**
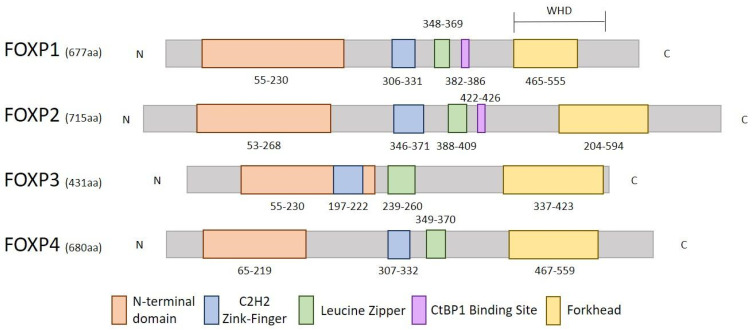
Structure and domains of FOXP proteins. The rectangles—protein domains; the numbers—amino acid numbers; WHD (Winged Helix Domain)—β-sheets + α-helices + wing-like loops; CtBP1—C-terminal binding protein 1 domain; forkhead domain—DNA-binding domain. The figure is a not-to-scale drawing.

All FOX transcription factors bind DNA through a conserved forkhead, or “winged helix,” domain composed of three α-helices and two wing-like loop structures [17]. In the FOXP subfamily, this DNA-binding domain is positioned at the C-terminal end, in contrast to most other FOX proteins, in which it is at the N-terminal end of the protein. In addition, the FOXP1 forkhead domain contains deletions within the wing regions compared with other FOX family members [18]. Unlike other forkhead subfamilies, FOXP proteins possess several conserved domains beyond the DNA-binding region [19,20]. These include a highly conserved C2H2 zinc finger domain, a leucine zipper motif, the winged helix domain (WHD) composed of β-sheets, α-helices, and wing-like loops resembling a helix–turn–helix structure [21], the forkhead DNA-binding domain, and the N-terminal domain [22]. Notably, FOXP1 and FOXP2 contain a C-terminal binding site for C-terminal binding protein 1 (CtBP1), which is absent in FOXP3 and FOXP4 [20,23,24,25] (Figure 1).

Whereas most FOX proteins function as monomers in solution and when bound to DNA [18], the ability of FOXP proteins to form heterodimers expands the functional repertoire of this subfamily [19,20]. Structural analyses have shown that the FOXP1 forkhead domain consists of five α-helices (H1–H5), three β-strands (S1–S3), and two wing regions (W1 and W2) [26,27]. FOXP2 and FOXP3 exhibit highly similar crystal structures, forming dimers analogous to FOXP1 but with distinct dimerization properties. Although the forkhead domains of all FOXP proteins enable C-terminal winged helix-mediated dimerization, FOXP3 dimers are considered more stable than those formed by FOXP1 or FOXP2 [24]. Mendoza et al. further identified both homo- and heterodimeric complexes, as well as higher-order oligomers comprising FOXP1, FOXP2, and FOXP4, in HEK293 cells and brain tissue [28].

FOXP proteins predominantly act as transcriptional repressors, and with the exception of FOXP3, they also contain polyglutamine tracts near their N-terminus [19,20]. Two independent subdomains contribute to the transcriptional repression activity of FOXP proteins. Subdomain 1 contains a highly conserved leucine zipper motif, similar to that of N-Myc, which mediates both homo- and heterodimerization among FOXP1, FOXP2, and FOXP4. Subdomain 2 includes a binding motif for the corepressor CtBP1 and is present in FOXP1 and FOXP2 but absent in FOXP4. In contrast, FOXP3 represses target gene expression through interactions with RUNX transcription factors [29,30].

FOXP1 is a transcription factor involved in multiple biological processes, including neural development, monocyte differentiation, macrophage function, T and B lymphocyte differentiation and T cells (Tregs) maintenance and differentiation [31,32,33]. It also contributes to the development of the heart, lungs, and central nervous system, with mutations causing FOXP1 syndrome, a neurodevelopmental disorder [31,34,35]. Multiple FOXP1 isoforms exist, with at least three expressed in adult lung and brain tissue and implicated in central nervous system development [19,20,36], while seven FOXP1 isoforms lacking part of the N-terminal (NH3-terminal) region have been identified, and three of these were detected in samples of Diffuse Large B-cell Lymphoma (DLBCL) [37] (Table 1).

The *FOXP2* gene is a key regulator of human language and cognitive development [38,39], influencing genes critical for nervous system function, particularly in brain regions involved in language and fine motor coordination [40]. It participates in embryonic development and cell-cycle regulation via pathways associated with neurogenesis, such as Wnt and Notch [41,42]. Variations in FOXP2 are linked to speech and language disorders, as well as a range of neuropsychiatric conditions [43,44,45]. There are six FOXP2 protein isoforms (Table 2).

**Figure 2:**
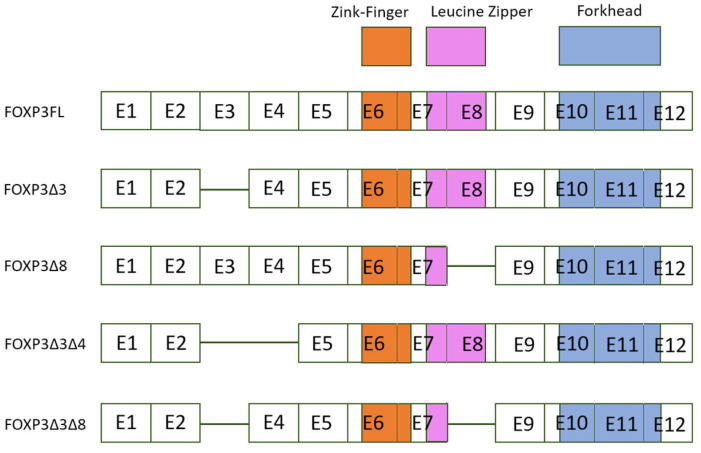
FOXP3 mRNA isoforms. E1–E12—exons; Straight line—exon is missing; The figure is a not-to-scale drawing.

FOXP3 is a key immune transcription factor primarily expressed in regulatory T (Treg) cells (CD4^+^/CD25^+^ or CD4^+^/CD25^−^) [46,47], cells that function as immunosuppressive lymphocytes [48], maintaining immune homeostasis and self-tolerance [22]. Treg cells, including natural (nTregs) and induced (iTregs) subsets, rely on FOXP3 for their development and function, making it a specific biomarker for this population [48,49,50]. FOXP3 is also found in other cell types, such as B lymphocytes and thymocytes, and in normal tissues like the lung, thymus, prostate, and breast [46,51,52,53]. In humans, five FOXP3 isoforms have been identified: FOXP3FL, FOXP3Δ3, FOXP3Δ3Δ4, FOXP3Δ8, and FOXP3Δ3Δ8 [52,53,54] (Figure 2).

FOXP4 is a transcription factor essential for development and organogenesis, particularly in the regulation of metabolic processes, immune function, and T-cell development [29,55]. It shares significant sequence similarity with *FOXP1* and *FOXP2* and, together, they form a multidomain transcriptional repressor complex [56]. FOXP4 is essential for maintaining Treg homeostasis and function and contributes to central nervous system development [57,58]. In the brain, it is co-expressed with FOXP1 and FOXP2 in regions such as the cortex, cerebellum, and striatum, where they may form heterodimers to regulate shared downstream target genes [59]. To date, fourteen FOXP4 protein isoforms have been identified (Table 3).

### 1.2. FOXP Genes and Cancer

FOXP-dependent cancer initiation and progression are associated with multiple hallmarks of malignancy, including immune evasion, resistance to growth suppressors, genomic instability and mutational burden, induction of angiogenesis, evasion of apoptosis, sustained proliferative signaling, and inflammation. Although accumulating evidence indicates that FOXP proteins can function either as oncogenes or tumor suppressors depending on cancer type [60,61], the molecular determinants and regulatory mechanisms that dictate whether FOXP proteins exert tumor-suppressive or oncogenic activities have yet to be fully elucidated.

*FOXP1* overexpression has been consistently associated with poor prognosis in several B-cell lymphomas, including DLBCL [62,63,64], primary cutaneous large B-cell lymphoma (PCLBCL) [65], follicular lymphoma [37], and gastric mucosa-associated lymphoid tissue lymphoma (MALT) [66], supporting its role as an oncogene in these malignancies. In these contexts, elevated FOXP1 expression is frequently correlated with worse clinical outcomes. These effects may arise from multiple molecular pathways, including arrest of the cell cycle at the G1/S transition and decreased phosphorylation of the retinoblastoma protein [67]. Other potential mechanisms involve nuclear interaction of FOXP1 with estrogen receptors α or β [68,69], suppression of MHC class II expression combined with activation of Wnt/β-catenin signaling [70,71], and chromosomal translocations driven by immunoglobulin heavy chain enhancer activity [61,72]. In contrast, FOXP1 is considered to have tumor-suppressive activity in certain epithelial malignancies, including lung, breast, prostate and pancreatic cancer. Takayama et al. observed that decreased *FOXP1* expression correlates with prostate cancer progression and poorer patient survival by modulating the androgen receptor and inhibiting cell proliferation and migration [73]. The *FOXP1* gene is located at chromosome 3p14.1, a region commonly described as a tumor suppressor locus; accordingly, loss of *FOXP1* expression has been associated with poor prognosis in breast cancer [74]. Moreover, FOXP1 can interact with FOXP3 through the nuclear factor of activated T cells (NFAT)–IL-2 promoter DNA complexes [74], indicating that its oncogenic or tumor-suppressive roles may be modulated by transcriptional networks and protein–protein interactions. Truncated FOXP1 isoforms are associated with DLBCL characterized by continuous nuclear factor kappa-light-chain-enhancer of activated B cells (NF-κB) activation, suggesting that these shorter forms disrupt the repressive function of full-length FOXP1 and enhance NF-κB signaling [37].

FOXP2 predominantly functions as a transcriptional repressor and has a context-dependent role in oncogenesis and cancer progression. FOXP2 suppresses the transcriptional activity of target genes through its zinc finger domain and can interact with CtBP1, a transcriptional corepressor that regulates the expression of tumor suppressor genes such as *BAX*, *PTEN*, and *CDKN2A* [75]. Through these interactions, FOXP2 has also been implicated in the repression of E-cadherin expression and the promotion of cellular invasion [60]. In addition, FOXP2 may modulate the expression of genes involved in key oncogenic signaling pathways, including IGF-1 (insulin-like growth factor 1), NF-κB, and Wnt signaling [76,77,78]. FOXP2 is downregulated in certain malignancies, such as breast, liver, and gastric cancers, whereas it is overexpressed in others [60,79,80,81], further supporting its dual functional role. Consistent with a tumor-suppressive function in specific contexts, Cuiffo et al. demonstrated that FOXP2 downregulation enhances tumor initiation in breast cancer, suggesting its role as a putative tumor and metastasis suppressor [82]. Similarly, *FOXP2* expression is reduced in hepatocellular carcinoma (HCC) tissues, where decreased expression correlates with poor overall survival and increased tumor invasiveness [83].

*FOXP3* is located on chromosome Xp11.23 [46], a region of particular biological significance due to its X-linked inheritance pattern. Consequently, a single genetic “hit” affecting *FOXP3* may be sufficient to disrupt its function and contribute to malignant transformation [53,84]. Genetic alterations in *FOXP3* include both single-nucleotide polymorphisms (SNPs) and microsatellite polymorphisms [52].Among the most frequently studied FOXP3 promoter polymorphisms are rs3761549 (C > T) and rs3761548 (C > A), both of which have been implicated in carcinogenesis [46]. Mutations and dysregulated expression of FOXP3 have been associated with immune dysfunction and tumor development [53].

Functionally, FOXP3 plays a central role in immune regulation and may contribute to tumor immune evasion [85]. Additionally, FOXP3 has been shown to inhibit angiogenesis through regulation of vascular endothelial growth factor (VEGF), as demonstrated in breast cancer MDA-MB-231 cell lines [86]. However, the prognostic significance of *FOXP3* expression in cancer is not uniform across tumor types. Elevated *FOXP3* expression is associated with poor prognosis in colorectal cancer [49], melanoma [52], and both non-small cell and small cell lung carcinoma [87], while higher *FOXP3* expression correlates with improved prognosis in breast, prostate and gastric cancers [52,53,88,89]. These divergent outcomes likely reflect tumor-type-specific functional roles and distinct molecular contexts in which FOXP3 is activated.

FOXP3 regulates transcription through both gene activation and repression [22]. The binding of FOXP3 to promoter and 5′ regulatory regions of *CTLA4* (encoding CTLA-4 receptor) and *IL2RA* (encoding CD25 and IL-2 receptor-α) induces histone acetylation and enhanced expression of CTLA-4 and CD25 in T cells. Conversely, FOXP3 binding to promoters of *IL-2*, *IL-7RA*, or *IFN-γ* is associated with reduced histone acetylation and chromatin remodeling, leading to transcriptional repression of these cytokines [53]. In breast cancer, FOXP3 has been characterized as a tumor suppressor [85,90,91,92], in part through transcriptional repression of oncogenes such as *HER2/ErbB2* [92] and *SKP2* [93]. Furthermore, FOXP3 interacts with several key transcription factors within the tumor microenvironment, including NF-κB, NFAT [94], and acute myeloid leukemia 1 (AML-1) [95], underscoring its role as a central regulator of immune and tumor-associated signaling networks.

The role of *FOXP4* in cancer remains relatively underexplored compared to other FOXP family members. FOXP4 regulates the expression of multiple genes involved in biological processes that contribute to cancer progression [96]. *FOXP4* was found to be overexpressed in A549 and H1703 non-small cell lung cancer (NSCLC) cell lines, and its depletion significantly inhibited cellular proliferation and invasion in these models [97]. Also, *FOXP4* has been linked to prostate cancer susceptibility in Chinese populations [98]. In addition, the long non-coding RNA FOXP4-AS1 has been identified as a potential poor prognostic biomarker in colorectal cancer and osteosarcoma [99], suggesting that FOXP4-related regulatory networks may influence tumor progression and clinical outcomes. FOXP4 regulates β-catenin expression by enhancing its transcription, thereby promoting the malignant progression of esophageal squamous cell carcinoma (ESCC) while also playing a significant role in tumor growth in the kidney and larynx [100]. *FOXP4* expression was reported to be significantly downregulated in renal cancers [29], implying that FOXP4 may also exert tumor-suppressive functions depending on the tissue context. These findings suggest that *FOXP4*, similar to other FOXP family members, may exhibit dual and context-dependent roles in tumorigenesis, although further studies are required to clarify its function.

Despite increasing evidence implicating FOXP family members in tumorigenesis, their roles in human carcinomas remain incompletely understood, particularly in the context of thyroid carcinoma. Given the context-dependent roles of FOXP proteins as either oncogenes or tumor suppressors, a comprehensive evaluation of their involvement in thyroid cancer is warranted. The potential relevance of FOXP transcription factors in thyroid cancer appears particularly important due to several distinctive biological characteristics of thyroid tumorigenesis. Thyroid cancer progression is strongly influenced by dysregulated MAPK, PI3K/AKT, Wnt/β-catenin, TGF-β, and immune-related signaling pathways, many of which are functionally interconnected with FOXP-associated regulatory networks. In addition, immune microenvironment modulation, epithelial–mesenchymal transition, radioiodine resistance, and dedifferentiation represent major determinants of aggressive thyroid cancer behavior, and increasing evidence suggests that FOXP family members may contribute to these processes. Unlike many solid tumors, thyroid cancer also exhibits a unique dependence on differentiation-associated pathways and sodium iodide symporter (NIS) regulation, which may further enhance the biological and clinical relevance of FOXP-mediated transcriptional programs in this malignancy. Therefore, this review aims to summarize current knowledge on FOXP transcription factors in thyroid cancer, examining their expression patterns, molecular mechanisms, clinical relevance, and therapeutic potential. To facilitate a clearer overview and critical evaluation of currently available evidence, key bioinformatic, transcriptomic, and TCGA-derived findings regarding FOXP family members in thyroid cancer are summarized in Table 4. By integrating bioinformatic, experimental, and clinical evidence, we aim to provide a comprehensive view of FOXP-driven cancer processes and highlightopportunities to implement these insights in potential clinical applications.

## 2. FOXP3 Gene in Thyroid Cancer

Among FOXP family members, *FOXP3* is the most studied gene in thyroid cancer. FOXP3, a transcription factor known for its role in regulatory T cells, is increasingly recognized as animportant player in thyroid cancer, particularly PTC, where it influences tumor progression, immune evasion, treatment resistance, and may serve as a prognostic marker and therapeutic target. The following sections explore these functions and their clinical significance.

### 2.1. FOXP3 Expression and Localization

*FOXP3* is expressed in a significant proportion of papillary thyroid carcinoma cases, and its elevated expression is more common in malignant thyroid tumors than in benign conditions like goiter, suggesting itscontribution to a more aggressive tumor phenotype [115]. In PTC, elevated *FOXP3* levels are linked to aggressive features such as lymph node metastases, extrathyroidal extension and multifocality, suggesting its contribution to a more aggressive tumor phenotype [116]. The aggressiveness of tumors depends not only on the level of *FOXP3* expression but also on its location within the cell. According to Cunha et al. FOXP3 is localized both in the cytoplasm and nuclei of cancer cells and in differentiated thyroid carcinoma-infiltrating lymphocytes [103]. In their study, nuclear localization of FOXP3 was associated with the aggressiveness of differentiated thyroid carcinomas. Furthermore, FOXP3-positive lymphocytes were more likely to infiltrate tumors smaller than 2 cm, without extrathyroidal invasion, and accompanied by chronic lymphocytic thyroiditis (LT) [103]. The same authors observed higher cytoplasmic FOXP3 immunostaining in malignant lesions compared with benign nodules and suggested that this localization might be related to increased mutation rates [103]. This finding is in agreement with previous reports showing that cytoplasmic FOXP3 arises from somatic mutations within theforkhead (FKH)/wingedhelix domain [117]. Cunha et al. also reported higher nuclear FOXP3 immunostaining in the younger patients and in the aggressive differentiated thyroid carcinomas with metastasis at diagnosis [103]. Ugolini et al. noticed predominant FOXP3 staining in the cytoplasm of thyroid tumor cells, with both nuclear and cytoplasmic staining in some cases of infiltrating cells [118], while Xin et al. reported that FOXP3 was mainly localized in the cytoplasm and nucleus of PTC cells and that its expression was associated with lymph node metastasis and TNM clinical stage [101].

### 2.2. FOXP3 and Immune Evasion in Thyroid Cancer

FOXP3 plays a key role in regulatory T-cell development and function, and in thyroid cancer, particularly PTC, its expression is linked to immune suppression that allows tumor cells to evade immunosurveillance [116]. FOXP3 induces a molecular mimicry and modulates the patterns of expression of various genes in cancer cells, which regulate immune response against thyroid tissue and favor an aggressive phenotype, but the specific function of FOXP3 in thyroid cancer cells is still unclear [90,102,104,105,119]. FOXP3-positive tumors exhibit a greater propensity for invasion and metastasis, potentially through the induction of immunosuppressive cytokines such as transforming growth factor-β1 (TGF-β1) and interleukin-10 (IL-10) [119]. This may reflect a form of molecular mimicry, whereby tumor cells adopt immunoregulatory features to suppress anti-tumor immune responses, thereby facilitating immune evasion and promoting tumor progression [119]. Hinz et al. demonstrated that *FOXP3* expression in pancreatic ductal adenocarcinoma cells was stimulated by TGF-β2, whereas TGF-β1 did not produce the same effect. Neutralizing TGF-β2 with specific antibodies partially reduced FOXP3 expression [119]. They observed a similar response when a constitutively active mutant of the TGF-β type I receptor/ALK5 was ectopically expressed. Furthermore, silencing *FOXP3* using small interfering RNA (siRNA) in pancreatic cancer cells led to increased expression of interleukin-6 (IL-6) and interleukin-8 (IL-8), indicating that FOXP3 acts as a negative regulator of transcription in these epithelial tumor cells. Since IL-6 expression is driven by NF-κB activity, and FOXP3 is known to interact with NF-κB in regulatory T cells (Tregs) to suppress cytokine gene transcription, they suggested that reducing FOXP3 levels may enhance IL-6 secretion by removing this inhibitory effect on NF-κB [119]. The finding that siRNA-mediated FOXP3 knockdown increased IL-6 expression supports the idea that FOXP3 functions in pancreatic cancer cells in a manner comparable to its role in Tregs. In addition, Hinz et al. determined that coculturing FOXP3-positive tumor cells with naïve T cells completely blocked T-cell proliferation without affecting T-cell activation. This antiproliferative effect was partially reversed when FOXP3 expression was specifically inhibited [119]. Their results suggest that pancreatic carcinoma cells can mimic the immunosuppressive properties of Tregs, potentially representing a novel mechanism by which pancreatic tumors evade immune surveillance. The same mechanism could potentially be responsible for molecular mimicry in thyroid tumors, but further research is necessary in order to confirm this. High levels of FOXP3 inhibit expression of PPARγ (nuclear hormone receptor) and caspase-3 (a key pro-apoptotic molecule) and increase expression of NF-ĸBand cyclin D1, which can lead to higher cell proliferation and migration, as well as hindering of apoptosis in cancer cells [85].

Previous studies reported that *FOXP3* expression in cancer cells was correlated with extrathyroidal invasion, infiltration of immune cells and distant metastasis [102,103,104,118]. Differences in *FOXP3* expression have also been observed between neoplastic and non-neoplastic tissues. In addition, patients whose tumors and tumor-infiltrating lymphocytes were positive for FOXP3 had shorter disease-free survival and overall survival compared with those who were negative for FOXP3 in both [105]. In thyroid cancer, the presence of Tregs is associated with aggressive disease. Patients with tumor-associated lymphocytes, particularly FOXP3-positive cells within tumor-involved lymph nodes, demonstrate increased disease progression as well as a higher incidence of invasion and metastasis [120]. Zeng et al. reported an absence of FOXP3^+^ Tregs in benign thyroid tumors, whereas a substantial accumulation of these cells was observed in the peritumoral regions of PTC. In patients with DTC complicated by chronic lymphocytic thyroiditis (CLT), the concurrent presence of CD68^+^, CD4^+^, CD8^+^, CD20^+^, FOXP3^+^ and Th17 lymphocytes within the tumor microenvironment was associated with lower tumor invasiveness, more favorable pathological characteristics, and improved prognosis [121]. However, the same study also demonstrated that among PTC patients with Hashimoto’s thyroiditis (HT), the rate of lymph node metastasis was significantly higher in those with high *FOXP3* expression in the tumor microenvironment compared with those exhibiting low *FOXP3* expression [121]. This finding suggests that elevated *FOXP3* expression may promote lymph node metastasis in PTC/HT patients [121]. Yang et al. reported that patients with HT exhibit reduced *FOXP3* expression and impaired Treg function, which may result from abnormal acetylation of FOXP3 [122]. Previous studies have indicated that *FOXP3* expression in PTC is not influenced by the coexistence of HT [104,123]. These findings indicate that *FOXP3* expression is not directly associated with the presence of HT;rather, the process of malignant transformation is likely responsible for the aberrant *FOXP3* expression observed in PTC cells [104,123]. Further, Zeng et al. found that the presence of Th17 cells in the tumor microenvironment of PTC/HT patients was associated with a better prognosis [124]. Accumulating evidence indicates that an imbalance between Th17 cells and Tregs is closely involved in the initiation and progression of multiple tumor types [125]. Zeng et al. proposed that external stimulation may rapidly induce Th17 differentiation while suppressing Treg-mediated inhibition, resulting in a shift in the Th17/Treg balance toward Th17 dominance [121]. Previous studies have shown that Th17 cells can inhibit lymph node metastasis and are associated with favorable outcomes in PTC with HT; therefore, a Th17-biased Th17/Treg balance may represent an important factor contributing to improved prognosis [124]. Within the tumor microenvironment shaped by both thyroid cancer and inflammation, Treg cells appear to promote lymph node metastasis, whereas Th17 cells may counteract the tumor-promoting effects of Tregs. A lower Th17/Treg ratio is associated with increased tumor invasiveness and metastatic potential, while a shift toward Treg dominance is linked to poorer prognosis. Nonetheless, the precise roles of Th17 and Treg cells in PTC lymph node metastasis require further experimental validation. Given that Th17 and Treg cells can interconvert under specific microenvironmental conditions, it remains unclear whether the observed immune alterations in PTC with HT are a cause of tumorigenesis or a consequence of tumor progression, warranting further investigation [121,124].

Previous studies have demonstrated that FOXP3 directly binds to the transcriptional start site of the chemokine receptor CXCR4, suggesting a regulatory role in *CXCR4* expression. CXCR4 and its ligand, CXCL12, are widely expressed across human cancers and drive key oncogenic processes, including tumor cell proliferation, invasion, and angiogenesis. Increasing evidence indicates that CXCR4–CXCL12 signaling contributes to the pathogenesis of PTC by promoting tumor growth, progression, and metastasis. Notably, *CXCR4* expression has been strongly associated with *BRAF* (serine/threonine-protein kinase B-Raf) mutation status and the degree of neoplastic infiltration [126]. These observations suggest that oncogenic BRAF activation may potentiate CXCR4-driven signaling pathways, thereby enhancing local tumor aggressiveness. It has therefore been proposed that cooperative interactions between *CXCR4* expression and *BRAF* mutation status contribute to an aggressive PTC phenotype [126]. A plausible mechanism underlying this cooperation is the recruitment and activation of inflammatory cells, including T lymphocytes and Tregs, which may further amplify CXCR4-mediated tumor-promoting signals within the tumor microenvironment.

### 2.3. Polymorphisms of FOXP3

Different studies reported high nuclear FOXP3 localization in papillary thyroid carcinoma and linked the rs3761548 polymorphism to cancer risk [115,127]. Jiang et al. examined the association of FOXP3 rs3761548 and rs2280883 polymorphisms with susceptibility to DTC in the Chinese Han population. They found that the minor allele A of rs3761548 was more frequent in DTC patients than in controls, while the major allele C of rs2280883 was less frequent in patients. Individuals with the rs3761548AC genotype had a higher DTC risk, whereas those with the rs2280883CT genotype had a lower risk. The AA/AC genotypes of the rs3761548 polymorphism were significantly more common in female than male DTC patients, whereas the rs2280883 CC/CT frequencies did not differ by sex [128]. In their study, Achilla et al. indicated that the A allele and the CA and AA genotypes of the rs3761548 genetic variant showed a big, statistically significant difference in their distribution between female PTC patients and controls [127]. This may help account for the higher prevalence of PTC in females than in males and could be related to *FOXP3* location on Xp11.23. Additionally, Jiang et al. reported that the rs3761548 AA/AC genotype was more frequent in more advanced tumors (diameter > 1 cm), while the rs2280883 CC/CT genotype was less common in those cases. Overall, rs3761548 AA/AC appears to be a potential risk genotype for DTC, while rs2280883 CC/CT is a protective genotype, suggesting these SNPs could indicate DTC aggressiveness [128]. Muayad et al. reported that, among patients with thyroid disorders, individuals carrying the heterozygous (AC) genotype of the rs3761548 polymorphism had significantly higher serum FOXP3 levels compared with those carrying the other genotypes [129].

### 2.4. Epigenetic Regulation of FOXP3Expression

*FOXP3* expression can be regulated by both methylation of DNA and miRNA. Achilla et al. analyzed the methylation status of the *FOXP3* gene in PTC patients. They reported no methylation in male and female controls regardless of rs3761548 genotype, nor in male PTC patients carrying either the A or C allele. In female PTC patients, an unmethylated status was observed only among those with the AA genotype. In contrast, methylation was identified in some females with the CC or CA genotypes, suggesting a possible interaction between *FOXP3* methylation status and rs3761548 genotypes in PTC susceptibility [127]. The combined analysis of *FOXP3* rs3761548 genotypes and methylation status indicated that methylated cytosines were detected only in female patients carrying the rs3761548 C allele [127]. Based on these findings, it was suggested by Achilla et al. that the C allele may be associated with methylation in female patients with CA or CC genotypes. In contrast, in healthy individuals carrying the CA or CC genotypes, both alleles were observed to be unmethylated [127]. The AA genotype of the rs3761548 polymorphism, which is located in the promoter region of the *FOXP3* gene, specifically the 5′-untranslated upstream region, has previously been associated with impaired *FOXP3* transcription, as the A allele has been shown to disrupt the binding of transcription factor [130]. Additionally, the A allele has been reported to be associated with reduced *FOXP3* expression, altered Treg function and cytokine secretion [131,132]. Accordingly, Achilla et al. proposed that the reduced *FOXP3* expression observed in PTC patients in relation to the rs3761548 genotype could be associated either with the presence of the A allele (AA genotype) or with methylation of the C allele in CA or CC genotypes. Furthermore, given that methylation was detected only in female patients, this observation was suggested to be potentially related to the higher incidence of PTC in females compared with males [127].

Previous studies have shown that *FOXP3* expression is upregulated, whereas miR-125b expression is downregulated in thyroid cancer tissues and thyroid cancer cell lines [133]. miR-125b suppresses the migration and invasion of anaplastic thyroid cancer cells by directly targeting PIK3CD. Wang et al. further demonstrated that miR-125b can inhibit *FOXP3* expression through direct binding to its 3′-UTR. In addition, they reported that overexpression of miR-125b enhanced the sensitivity of thyroid cancer cells to cisplatin by inducing autophagy via the Atg7 pathway, both in vitro and in vivo [133]. Discrepancies in *FOXP3* expression reported in different thyroid cancer studies may be attributed to differences in the distribution of male and female participants, variations in FOXP3 gene polymorphisms, and the presence of distinct miRNAs.

### 2.5. FOXP3 as a Therapeutic Target and PTC Biomarker

FOXP3 is a promising therapeutic target due to its roles in immune evasion and therapy resistance. Approaches that target FOXP3+ Tregs or modulate theirexpression in tumor cells could boost anti-tumor immunity and improve treatment outcomes [116,118]. Vaccination against FOXP3-expressing cells has also been shown to enhance tumor immunity, highlighting a potential new approach for cancer immunotherapy [118]. However, FOXP3’s function is complex, while it often correlates with poor prognosis in thyroid cancer, in some cancers, its upregulation can have anti-tumor or anti-metastatic effects, emphasizing the need for further research to clarify its role and to develop effective therapeutic strategies. After immunohistochemistry, Neves Junior et al. and Mohamed et al. observed high positivity for FOXP3 in PTC cells, which led them to conclude that FOXP3 could potentially be used as a biomarker for PTC diagnosis [103,104,123]. On the other hand, Ugolini et al. observed lower FOXP3 staining in PTC cells [118]. We can conclude that in order to use FOXP3 as a diagnostic marker, more research is needed.

**Figure 3:**
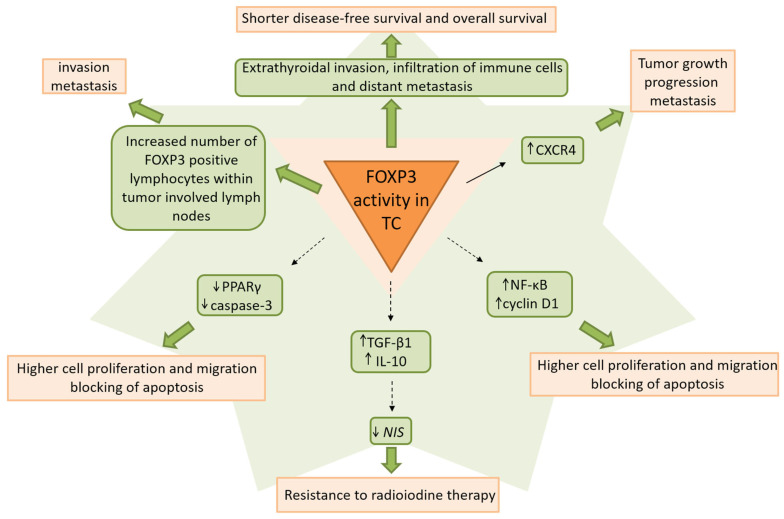
FOXP3 in thyroid cancer: Oncogenic role of FOXP3 in thyroid cancer progression. FOXP3 primarily acts as an oncogene in thyroid cancer. It upregulates the expression of NF-κB, cyclin D1, and CXCR4, while downregulating PPARγ and caspase-3. In addition, FOXP3 stimulates TGF-β and IL-10 signaling pathways. A higher number of FOXP3-positive lymphocytes in tumor-involved lymph nodes, along with increased FOXP3 activity in thyroid cancer, contributes to tumor progression, resistance to radioiodine therapy and reduced overall survival. Tumor progression is characterized by enhanced cell proliferation and migration, suppression of apoptosis and increased invasiveness. Solid lines: Experimentally validated in thyroid cancer, dashed lines: inferred from non-thyroid tumor models.

FOXP3, a key transcription factor in Tregs, plays a central role in PTC by promoting tumor progression, immune evasion, and therapy resistance through Treg activation and cytokine modulation (Figure 3). Its high expression and cytoplasmic localization are linked to aggressive features and reduced radioiodine sensitivity, while genetic polymorphisms and epigenetic regulation affect its expression. Given these roles, FOXP3 represents both a potential therapeutic target and a biomarker for PTC, although further research is needed to clarify its complex, context-dependent functions. The reported variability in *FOXP3* expression across studies, including findings of both increased and decreased expression, may be influenced by differences in the FOXP3 isoforms expressed among various cell types, subcellular localization of FOXP3, the Treg and tumor cell populations examined, the methods of analyses, sample sizes, and tumor location and stage [101,102,104,105,116,133,134].

Despite its potential therapeutic relevance, targeting FOXP3-associated pathways presents several important challenges and limitations. FOXP3 is a key regulator of regulatory T-cell (Treg) differentiation, immune homeostasis, and peripheral immune tolerance; therefore, systemic inhibition of FOXP3 activity could potentially disrupt normal immune regulation and increase the risk of autoimmune and inflammatory complications [99,100,110]. In addition, FOXP3 exhibits context-dependent functions and may exert both tumor-promoting and tumor-suppressive effects depending on the cellular context, tumor subtype, and microenvironmental conditions [58,99,103]. Another important limitation is the difficulty in selectively targeting tumor-associated FOXP3 signaling without affecting physiological Treg-mediated immune functions [110,111].

Furthermore, transcription factors are generally considered challenging therapeutic targets due to their complex protein–DNA interactions, lack of well-defined drug-binding pockets, and broad involvement in essential physiological regulatory networks [135,136]. Consequently, future therapeutic approaches will likely require more selective modulation of FOXP3-associated pathways or tumor-specific downstream regulatory mechanisms rather than complete systemic FOXP3 inhibition.

## 3. FOXP4 in Thyroid Cancer

In recent years, FOXP4 has also emerged as a potential regulator of thyroid cancer progression. Accumulated studies have provided results that demonstrate its oncogenic function. FOXP4 facilitates thyroid cancer progression by activating key oncogenic pathways and influencing the tumor microenvironment, resulting in a more aggressive TC phenotype, and its tumor-promoter roles will be discussed in this section.

### 3.1. FOXP4 Expression Patterns

Despite limited data on *FOXP4* expression in various malignant diseases, several studies have shown elevated *FOXP4* expression in thyroid cancer, particularly in papillary thyroid carcinoma [106]. Zhou et al. performed TCGA-based transcriptomic analyses and demonstrated significantly increased FOXP4 mRNA expression in thyroid cancer tissues compared with normal thyroid tissues [106]. Elevated FOXP4 expression was further associated with aggressive clinicopathological characteristics, including extrathyroidal invasion, vascular invasion, and distant metastasis, supporting its potential role as a marker of tumor aggressiveness [106,107]. Functional enrichment and network analyses additionally linked FOXP4-associated genes to pathways involved in proliferation, migration, immune regulation, and epithelial–mesenchymal transition [106]. The same authors then confirmed FOXP4 overexpression in TC tissue samples and cell lines through immunohistochemistry and quantitative PCR [106,107]. The agreement between in silico and experimental data suggests elevated *FOXP4* expression is a robust molecular feature in thyroid pathogenesis, although the study by Zhou et al. 2024 should be interpreted with caution, as an expression of concern has been issued by the journal regarding the publication. Additionally, the predominant nuclear localization of FOXP4 in thyroid cancer tissues supports its direct involvement in regulating TC progression [106,107]. However, further research is needed on FOXP4 expression in specific TC subtypes, as the available literature primarily addresses papillary thyroid carcinoma.

### 3.2. FOXP4 Regulatory Networks

Functional studies on thyroid tumor cell lines have shown that elevated *FOXP4* expression promotes tumor cell proliferation, migration, and epithelial–mesenchymal transition, primarily by regulating tumor-suppressive pathways [106,107]. Conversely, *FOXP4* knockdown induces cell-cycle arrest and less aggressive behavior in TC cells [106,107].

At the molecular level, FOXP4 appears to regulate multiple oncogenic pathways through both direct transcriptional regulation and indirect downstream signaling effects. Previous studies suggest that FOXP4 may transcriptionally enhance β-catenin expression rather than directly interacting with β-catenin protein itself [100]. Through activation of Wnt/β-catenin signaling, FOXP4 promotes the expression of downstream oncogenic targets, including c-Myc and cyclin D1, thereby contributing to increased proliferation, migration, and epithelial–mesenchymal transition [100,108]. In addition, FOXP4 may indirectly modulate Akt/mTOR and TGF-β-associated signaling networks through interactions with downstream regulatory molecules, including the tumor suppressor FBXW7 [106,107,108,137,138,139,140,141].

FOXP4-mediated oncogenic activity is additionally influenced by post-transcriptional regulatory mechanisms. The FOXP4-AS1/miR-4525 regulatory axis contributes to FOXP4 overexpression by suppressing microRNA-mediated inhibition of FOXP4 translation, thereby promoting thyroid cancer progression [106,137,142,143]. Moreover, evidence from non-thyroid tumor models indicates that FOXP4 may participate in broader transcriptional programs involved in invasion, migration, and EMT-related signaling [108,141]. However, several of these mechanistic observations have not yet been fully validated in thyroid cancer-specific experimental systems, and additional studies are required to distinguish direct FOXP4 transcriptional targets from indirect downstream effects.

*FOXP4* is also involved in a competing endogenous RNA (ceRNA) network with FOXP4-AS1 long non-coding RNA and miR-4525, allowing for precise post-transcriptional regulation of its expression [106,137,142,143]. Through sequestration of miR-4525, FOXP4-AS1 inhibits microRNA-mediated suppression of *FOXP4*, resulting in increased FOXP4 protein levels. These observations are consistent with evidence that microRNAs and long non-coding RNAs play significant roles in thyroid cancer progression and in the regulation of the FOX gene family [144,145,146].

**Figure 4:**
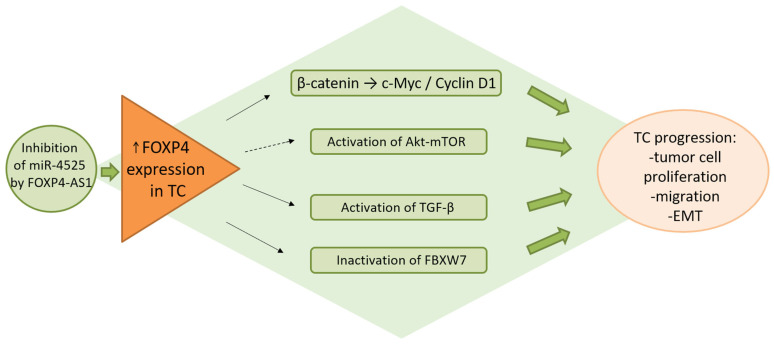
FOXP4 in thyroid cancer: Oncogenic role of FOXP4 in thyroid cancer progression. FOXP4 functions predominantly as an oncogene in thyroid cancer. Its expression is upregulated through inhibition of miR-4525 by FOXP4-AS1, leading to activation of key oncogenic pathways, including Wnt/β-catenin, Akt–mTOR, and TGF-β signaling, along with inactivation of the tumor suppressor FBXW7. These molecular events collectively promote tumor progression, characterized by increased proliferation, migration, and epithelial–mesenchymal transition (EMT). Solid lines: Experimentally validated in thyroid cancer, dashed lines: inferred from non-thyroid tumor models.

Taken together, these findings show that *FOXP4* plays a central role in controlling proliferative, migratory, and microenvironmental signaling networks (Figure 4). This supports its importance in aggressive thyroid cancer and suggests it could be useful for developing prognostic biomarkers or targeted therapies.

## 4. FOXP2 in Thyroid Cancer

Compared to *FOXP3* and *FOXP4*, *FOXP2* has a more complex and variable expression pattern in thyroid cancer. FOXP2 was first recognized for its role in speech and language development. Recent studies show that FOXP2 is involved in various malignancies. Its role can shift between oncogene and tumor suppressor, depending on tumor type and cellular environment [147,148,149]. Transcriptomic and network-based analyses further suggest that FOXP2-associated gene signatures are linked to pathways involved in apoptosis, cellular differentiation, stromal signaling, and immune-related interactions, although the currently available evidence in thyroid cancer remains limited and partially context-dependent [109,111,112,113,128].

### 4.1. FOXP2 Expression Patterns

In thyroid cancer, *FOXP2* expression is often disrupted and varies depending on the tissue-specific context. Most studies report lower FOXP2 expression in cancerous thyroid tissue compared to normal thyroid tissue. This suggests a tumor-suppressor role [150,151]. The decrease in *FOXP2* can result from post-transcriptional mechanisms of gene expression regulation, including microRNA regulation [152]. For example, miR-221-3p can lower *FOXP2* levels via the Hedgehog signaling pathway. This leads to increased cell growth and decreased cell death [151]. Transcriptomic and network analysis reveal that *FOXP2* is involved in complex gene networks, and these findings expand its roles in tumorigenesis [109,110,111]. Recent single-cell RNA sequencing data show that *FOXP2* expression can vary across different parts of the tumor and surrounding tissue, underscoring the importance of its spatial effects [144]. Overall, this data indicates that *FOXP2* expression patterns reflect a combination of transcriptional, post-transcriptional, and microenvironmental regulatory mechanisms in TC, highlighting its complex and still unexplored function.

### 4.2. FOXP2 Regulatory Networks

*FOXP2* plays an important role in regulating key cellular processes in thyroid cancer, particularly proliferation and apoptosis. Tumor-suppressive function was demonstrated in functional studies, which revealed that elevated *FOXP2* inhibits tumor cell proliferation and induces apoptosis [150]. These effects are mediated via transcriptional regulation of downstream targets and modulation of oncogenic signaling pathways. Similar tumor-suppressive roles of *FOXP2* have been reported in other malignancies, reinforcing its role as a regulator of tumor growth dynamics [151].

At the molecular level, *FOXP2* exerts its effects by directly regulating specific target genes. Yang et al. observed that RPS6KA6 is transcriptionally activated by FOXP2, and that, when overexpressed, *FOXP2* inhibits cell proliferation and promotes apoptosis in thyroid cancer [4]. Restoration experiments further confirmed that the FOXP2–RPS6KA6 axis plays a key role in controlling thyroid cancer cell proliferation and apoptosis [150]. This regulatory axis is closely linked to the PI3K/AKT signaling pathway, a central regulator of cell survival and proliferation that is frequently dysregulated, further suggesting that FOXP2 is a potential prognostic biomarker in thyroid cancer [150].

In addition, *FOXP2* modulates the Wnt/β-catenin signaling pathway, influencing proliferation, migration, and apoptosis of thyroid cancer cells [112]. *FOXP2* is also involved in Hedgehog signaling, where microRNA-mediated suppression contributes to tumor cell proliferation and survival [151].

Beyond direct effects on tumor cells, FOXP2 also impacts the tumor microenvironment. Studies have shown that *FOXP2* expression correlates with immune cell infiltration patterns in thyroid cancer, alluding to an immunomodulatory function [102]. Single-cell transcriptomic analyses show that FOXP2 can activate *PROS1* transcription in cancer-associated fibroblasts. PROS1 then triggers the MERTK/WNT/TGF-β signaling axis. This promotes tumor progression through paracrine tumor–stroma interactions [113]. These findings also support FOXP2’s indirect role in promoting thyroid cancer aggressiveness and its multilayer function in TC.

Beyond its direct effects on tumor cell proliferation and apoptosis, FOXP2 may also contribute to broader transcriptional and epigenetic regulatory programs relevant to thyroid cancer progression. Increasing evidence from multiple solid tumor models suggests that FOXP2 activity is strongly context-dependent and may be influenced by tissue-specific transcriptional networks, microenvironmental conditions, alternative splicing events, and interactions with non-coding RNAs. Although mechanistic studies in thyroid cancer remain limited, FOXP2 has been linked to pathways involved in epithelial–mesenchymal transition, cellular differentiation, and treatment resistance in several malignancies. These observations suggest that FOXP2 may function not only as a classical tumor suppressor, but also as a context-sensitive regulator whose biological effects depend on the molecular landscape of individual tumors [60,76,77,78,82,83].

In addition, the prognostic and functional significance of FOXP2 expression in thyroid cancer may be influenced by intratumoral heterogeneity and differences in cellular composition within the tumor microenvironment. Recent transcriptomic and single-cell studies indicate that FOXP2-associated signaling may differ between tumor cells, stromal compartments, and immune-associated populations, further complicating interpretation of its biological role [109,110,111,113,144]. Therefore, future studies should aim to clarify the direct transcriptional targets of FOXP2 in thyroid cancer, distinguish tumor-cell-specific from microenvironment-related effects, and determine whether distinct FOXP2 isoforms exert differential oncogenic or tumor-suppressive functions [60,113,144].

**Figure 5:**
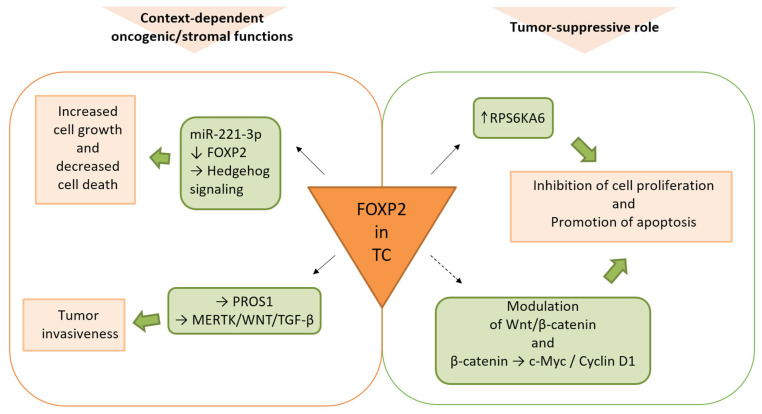
FOXP2 in thyroid cancer. Dual role of FOXP2 in thyroid cancer: context-dependent oncogenic and tumor-suppressive functions. FOXP2 exhibits a context-dependent role in thyroid cancer, acting as both an oncogene and tumor suppressor. On the oncogenic side, reduced FOXP2 expression mediated by miR-221-3p via the Hedgehog pathway promotes cell proliferation, decreases apoptosis, and enhances tumor invasiveness through activation of PROS1 and MERTK/Wnt/TGF-β signaling. Conversely, FOXP2 exerts tumor-suppressive effects by inhibiting Wnt/β-catenin signaling, leading to decreased expression of β-catenin, c-Myc, and cyclin D1, increased RPS6KA6 expression, reduced cell proliferation, and enhanced apoptosis. Solid lines: Experimentally validated in thyroid cancer, dashed lines: inferred/transcriptomic/single-cell evidence.

On the whole, FOXP2 functions as a context-dependent regulator in thyroid cancer biology, integrating transcriptional control of cell-cycle regulators, modulation of key signaling pathways, and interacting with the tumor microenvironment (Figure 5). While its predominant role appears tumor-suppressive at the cellular level, its involvement in stromal signaling and treatment resistance highlights multiple contributions to thyroid cancer progression, which need further investigation.

## 5. FOXP1 in Thyroid Cancer

There is currently limited evidence regarding the role and expression of the *FOXP1* gene in thyroid cancer [114]. Agbektas et al. analyzed differences in the expressionof the *FOXP1* gene in patients with DTC before and after radioactive iodine therapy. The authors reported significantly elevated *FOXP1* gene expression in patients with DTC prior to treatment compared with controls. Moreover, *FOXP1* expression differed significantly after treatment relative to pretreatment levels. However, FOXP1 protein levels were higher in the control group. Therefore, despite increased *FOXP1* mRNA expression in DTC before and after RAI treatment, FOXP1 protein expression was decreased, indicating a discordance between mRNA and protein levels. In healthy controls, FOXP1 protein levels were higher than in DTC patients. The discrepancy between *FOXP1* mRNA and protein expression indicates that post-transcriptional regulatory mechanisms, such as miRNA-mediated repression, impaired mRNA translation, or altered protein stability and degradation, may modulate FOXP1 protein levels independently of gene transcription, potentially limiting the utility of mRNA expression alone as a biomarker. Finally, they concluded that FOXP1 acts as a tumor suppressor by inhibiting the proliferation of DTC cells [114].

Abnormal expression of this gene was previously reported in various types of cancer [74,153,154,155,156,157,158,159]. Banham et al. were the first to report overexpression of the *FOXP1* gene in DLBCL, as well as its abnormal expression in various solid tumors [153]. Barranset al. reported higher expression of the *FOXP1* gene in a subset of DLBCL, in the non-germinal center activated B-cell type with expressed *BCL2* and absence of t(14;18) [63]. Wen et al. demonstrated that *FOXP1* gene expression was increased in acute myeloid leukemia (AML) and was a promising biomarker for predicting responses to immunotherapy in AML patients. They also found that the methylation level in the *FOXP1* gene was significantly lower among samples from AML patients compared to healthy controls and that methylation of *FOXP1* reduced the level of FOXP1 mRNA, which indicates that DNA methylation regulates *FOXP1* gene expression. They also suggested that FOXP1 modulates the tumor immune microenvironment by controlling immune cell infiltration in AML [158].

In various cancer types, such as pancreatic, colon, stomach, head, neck, lung, endometrial, renal, prostate, and breast cancers, FOXP1 can function as a tumor-suppressor [68,153,157,159,160,161], while, in other cancer types such as HCC, DLBCL and MALT type of marginal zone B-cell lymphoma, it can function as an oncogene [72,74,154,158,161]. Shorter isoforms of these proteins function as oncogenes, while wild-type FOXP1 functions as a tumorsuppressor. Depending on cancer type, overexpression of *FOXP1* is associated with good or bad disease progression [161]. In lymphomas, decreased expression of *FOXP1* is associated with favorable disease outcome and overall survival, but in solid tumors, this decreased expression is an unfavorable prognostic factor. Jiang et al. found that *FOXP1* expression in MALT lymphoma with large cells was higher than in MALT lymphoma without large cells and that most of the large tumor cells were positive for *FOXP1* expression. They also found a higher *FOXP1* expression rate in the high clinical stage, as well as that the group positive for *FOXP1* expression had a higher cell proliferation index. Finally, they concluded that FOXP1 expression may enhance the proliferation and invasion of tumor cells, leading to a worse clinical outcome [155]. Though in solid tumors, if FOXP1 is located in the nucleus, its decreased expression is an indicator of good overall survival. Additionally, in patients with solid tumors, decreased *FOXP1* expression is also correlated with an unfavorable relapse-free survival [156]. In their study, Sheng et al. demonstrated that expression of *FOXP1* was decreased in lung adenocarcinoma tissue, as well as that FOXP1 can potentially promote apoptosis and prevent tumor cell growth via its regulation of expression of various genes involved in chemokine signaling pathways such as *CCR1*, *ADCY5*, *GNG7*, *VAV3* and *PLCB1* [157]. Evidence from Wang et al. suggests that FOXP1 has a suppressive role in pancreatic tumor development [159]. Patients exhibiting high *FOXP1* expression showed a survival advantage compared with those with lower expression levels. Experimental models confirmed that increased *FOXP1* expression significantly limited pancreatic cancer growth both in vivo and in vitro, while decreased expression contributed to enhanced tumor formation. Further mechanistic investigation revealed that FOXP1 binds directly to the promoter region of *IRF1*, leading to activation of its transcription [159]. Because Xiao et al. in their metadata analysis included information about *FOXP1* expression in breast cancer, endometrial cancer, hepatocellular carcinoma, NSCLC, prostate cancer, colorectal cancer, epithelial ovarian cancer, and neuroblastoma, they considered that their finding may be applicable to all solid tumors [156].

Yang et al. reported that *FOXP1* is overexpressed in mucinous minimal deviation adenocarcinoma MDA of the uterine cervix and that in their samples, FOXP1 was localized in the cytoplasm of cancer cells, which was also reported by Giatromanolakiet al. [160,162]. Predominantly, FOXP1 is localized in the nucleus [153]. One of the possible mechanisms by which FOXP1 changes localization is protein phosphorylation [163].

In summary, evidence regarding FOXP1 in thyroid cancer remains limited. Although increased *FOXP1* gene expression has been reported in DTC, reduced protein levels suggest complex regulation beyond transcriptional control. Studies in other cancers demonstrate that FOXP1 function is highly context-dependent and influenced by isoform expression, subcellular localization, and epigenetic or post-transcriptional regulatory mechanisms. Therefore, additional studies are required to clarify the molecular mechanisms regulating FOXP1 in thyroid cancer and to determine its diagnostic, prognostic, and therapeutic relevance.

## 6. Clinical Significance and Prognostic Implications

### 6.1. Associations with Tumor Aggressiveness

FOXP transcription factors, especially *FOXP3* and *FOXP4*, are strongly associated with aggressive clinicopathological features in thyroid cancer. These patterns suggest FOXP expression could be a biomarker for high-risk patients. Such patients may benefit from more intensive treatment or surveillance.

*FOXP3* gene expression correlates with several indicators of tumor aggressiveness. Cunha et al. reported an association between *FOXP3* gene expression and aggressive differentiated thyroid carcinomas, including larger tumor size and advanced stage [103]. Multiple studies confirm this, including Mohamed et al., who found *FOXP3* in PTC is linked to higher tumor stage, extracapsular extension, and higher radioiodine doses [104]. *FOXP4* gene expression also correlates with aggressive features of TC. Zhou et al. showed that high expression is linked to poor prognosis, extrathyroidal invasion, vascular invasion, and distant metastasis in thyroid cancer [106,107]. These consistent findings across cohorts and populations support FOXP3 and FOXP4 as potentialmarkers for aggressive disease.

Tumor size is a key prognostic factor in thyroid cancer, and larger tumors generally have worse outcomes [164,165]. *FOXP3* expression is consistently linked with larger tumor size in several studies [103,104]. This could imply that FOXP3 helps drive tumor growth. Alternatively, *FOXP3* expression might rise as tumors increase due to hypoxia, metabolic stress, or other microenvironmental changes. Extrathyroidal extension represents tumor invasion beyond the thyroid capsule into surrounding tissues, which is a critical prognostic factor that influences staging and treatment [166]. Both *FOXP3* and *FOXP4* expression correlate with extrathyroidal extension [103,104,106], indicating that FOXP factors may facilitate tumor invasion by indirectly degrading the extracellular matrix, affecting cell adhesion, and promoting migration.

### 6.2. Lymph Node and Distant Metastasis

Lymph node metastasis occurs in about 30–80% of papillary thyroid carcinoma patients, and this rate depends on the extent of surgical dissection and pathological examination [167]. Lymph node metastasis in PTC does not greatly lower overall survival. However, it increases the risk of recurrence and may require more aggressive treatment. FOXP expression patterns show clear links with lymph node metastasis. Mohamed et al. found that *FOXP3* expression in PTC was significantly associated with lymph node metastasis [104]. This association remained significant in multivariate analysis, adjusting for other clinicopathological variables, suggesting that *FOXP3* expression provides independent prognostic information. The mechanisms by which FOXP3 promotes lymph node metastasis likely include enhanced migration and invasion, as well as potential effects on lymphangiogenesis. Zeng et al. specifically studied FOXP3 in Hashimoto’s thyroiditis. They reported that FOXP3 promotes lymph node metastasis in PTC patients with HT [121]. This finding is interesting because PTC with HT is generally thought to have a better prognosis. The observation that FOXP3 expression associates with lymph node metastasis, even in this favorable context, suggests FOXP3 is a robust marker of metastatic potential.

Distant metastasis, usually to the lungs and bones, occurs in about 5–10% of differentiated thyroid cancer patients, which significantly worsens the prognosis [168]. Previous studies noted that high *FOXP4* expression is connected with distant metastasis in thyroid cancer [106,107]. Similarly, Mohamed et al. found an association between *FOXP3* expression and distant TC spreading [104]. These findings suggest that FOXP factors may help drive all steps of the metastatic process, from local invasion to the spread and colonization of distant organs.

### 6.3. Treatment Resistance

FOXP3 could be a marker for radioiodine resistance, which is a major clinical problem linked to poor outcomes. In PTC, *FOXP3* expression has been linked to resistance to radioiodine therapy, a standard treatment for the disease [104,118]. Ma et al. demonstrated that sodium iodide symporter (NIS) transcript levels were markedly reduced in PTC compared with normal thyroid tissue [169]. Immunohistochemical analysis further revealed diminished NIS protein expression in tumor tissues, with residual NIS predominantly localized to the cytoplasm rather than the plasma membrane. These findings indicate that both NIS expression and subcellular localization are profoundly altered in malignant thyroid tissue. Notably, *FOXP3* expression was correlated with decreased *NIS* expression, suggesting a potential inhibitory role for FOXP3 in NIS regulation. Ma et al. showed that TGF-β induces *FOXP3* expression and proposed that FOXP3, in turn, promotes TGF-β1 secretion, thereby establishing a positive feedback loop that sustains *FOXP3* expression [169]. Collectively, thisdata supports a model in which FOXP3-driven TGF-β1 signaling represses *NIS* expression, contributing to resistance to radioiodine therapy in PTC. On the other hand, BRAF also inhibits *NIS* expression, and *BRAF* mutation is one of the most prevalent mutations in thyroid cancer [170]. These findings suggest that factors other than TGF-β1 may also mediate repression of NIS, and that *BRAF* mutation status may substantially contribute to this effect. Moreover, given that FOXP3 functions as a transcription factor, it is plausible that FOXP3 directly regulates *NIS* expression through binding to its transcriptional regulatory regions. Redifferentiation agents, such as selumetinib (a MEK inhibitor), can restore NIS expression in some patients [171].

FOXP2 has also been implicated in mechanisms of therapeutic resistance. In BRAF V600E-mutant papillary thyroid cancer, silencing FOXP2 has been shown to reverse resistance to the BRAF inhibitor vemurafenib [172]. This observation suggests that FOXP2 contributes to adaptive resistance pathways, potentially by modulating survival signaling networks. The context-dependent role of FOXP2 illustrates the complexity of its function in thyroid cancer. FOXP-positive, radioiodine-resistant patients might also need other options, such as tyrosine kinase inhibitors, immunotherapy, or targeted drugs, and alternative therapeutic approaches need further investigation in this context.

Collectively, these findings suggest that FOXP family members may have potential therapeutic relevance in thyroid cancer through several mechanisms, including modulation of tumor immune responses, regulation of radioiodine sensitivity, and interaction with oncogenic signaling pathways. In particular, FOXP3-associated immune evasion and suppression of NIS expression indicate possible roles in resistance to conventional therapies, while FOXP2 and FOXP4 appear to influence signaling networks involved in tumor progression and treatment adaptation. Although direct FOXP-targeted therapies are currently unavailable, emerging approaches aimed at modulating FOXP-associated pathways, including immune-targeted therapies, epigenetic modulation, RNA-based strategies, and pathway-specific inhibitors, may represent promising future therapeutic directions in aggressive and radioiodine-refractory thyroid cancer.

### 6.4. Prognostic Value and Survival Outcomes

Recent studies have examined links between FOXP expression and survival in thyroid cancer patients. The results vary depending on the specific FOXP family member, patient group, and follow-up period.

FOXP4 is linked with poor prognosis in thyroid cancer. Zhou et al. found that high *FOXP4* expression is associated with worse prognosis in thyroid cancer patients [106,107].Although robust disease-free survival analyses specifically evaluating FOXP4 expression in thyroid cancer remain limited, elevated FOXP4 expression has consistently been associated with clinicopathological characteristics linked to increased recurrence risk, including extrathyroidal invasion, vascular invasion, and distant metastasis [106,107]. Furthermore, TCGA-based analyses of the FOXP4-AS1 regulatory axis demonstrated associations with disease-free interval and tumor aggressiveness in papillary thyroid carcinoma, further supporting the potential prognostic relevance of FOXP4-related signaling networks [142]. Collectively, these findings suggest that dysregulation of the FOXP4 signaling axis may contribute to unfavorable disease outcomes; however, large prospective studies with standardized survival analyses are still required to validate its independent prognostic significance.

The prognostic value of FOXP3 has been studied in several reports, with variable findings. Cunha et al. found that *FOXP3* expression is associated with aggressiveness in differentiated thyroid carcinomas [103]. This suggests a worse prognosis, while Mohamed et al. reported that FOXP3 expression correlated with worse prognostic variables, such as CK19 (cytokeratin 19), which showed a stronger correlation with 10-year overall survival [104]. Whether FOXP3 provides independent prognostic information remains to be determined, as its interaction with other markers requires further study.

The context of Hashimoto’s thyroiditis also influences prognostic associations. Mohamed et al. found that HT with PTC was associated with better prognostic variables, including smaller tumor size and lower metastasis rates, suggesting a protective role of the autoimmune background [104]. However, Zeng et al. reported that *FOXP3* expression promotes lymph node metastasis even in PTC patients with HT [121], suggesting that *FOXP3* expression may override the generally favorable prognosis associated with HT.

When considering recurrence, this represents an important outcome in thyroid cancer, as recurrent disease requires additional treatment and is associated with worse long-term outcomes. Li et al. identified specific FOXP3+ Treg cell subsets associated with recurrence in papillary thyroid carcinoma using high-dimensional flow cytometry [173]. This finding suggests that not only *FOXP3* expression in tumor cells but also the characteristics of FOXP3+ immune cells in the tumor microenvironment influence recurrence risk.

Although currently available prognostic studies remain limited and heterogeneous, accumulating evidence suggests that dysregulated FOXP expression may contribute to clinically aggressive thyroid cancer phenotypes and unfavorable patient outcomes.The potential of FOXP factors as prognostic biomarkers must be validated in large, prospective cohorts with long-term follow-up. Ideally, prognostic models incorporating FOXP expression alongside established clinicopathological variables (age, tumor size, lymphatic and vascular invasion, extrathyroidal extension, lymph node metastasis, distant metastasis) and molecular markers (BRAF mutation, TERT promoter mutation) should be developed and validated. Such integrated models could improve risk stratification and guide personalized treatment decisions.

To further explore the potential prognostic relevance of FOXP factors in thyroid cancer, we performed an exploratory analysis using publicly available data from the cBioPortal Thyroid Carcinoma cohort (TCGA, Firehose Legacy; n = 509) [174,175,176]. Receiver operating characteristic (ROC) analysis demonstrated overall limited discriminative performance of individual FOXP markers for metastasis. However, FOXP4 expression (AUC = 0.593, *p* = 0.035) and FOXP2 methylation (AUC = 0.604, *p* = 0.018) showed statistically significant associations, while FOXP2 expression demonstrated an inverse association with metastasis (AUC = 0.407, *p* = 0.034). More importantly, FOXP2 methylation emerged as a strong predictor of extrathyroidal extension (ETE) (AUC = 0.786, *p* < 0.001), whereas FOXP2 expression again showed an inverse association (AUC = 0.339, *p* = 0.017). Similar findings were observed for lymph node involvement (AUC = 0.638, *p* < 0.001). Furthermore, FOXP2 expression negatively correlated with its methylation status (r = −0.570, *p* < 0.001), supporting a potential methylation-driven silencing mechanism underlying tumor invasiveness. Collectively, these findings further support the prognostic relevance of FOXP2 in thyroid cancer progression and local invasiveness, although additional experimental and clinical validation is required.

## 7. Conclusions and Future Perspectives

In conclusion, FOXP transcription factors form a complex, as well as context-dependent network that strongly affects thyroid cancer. FOXP3 and FOXP4 are mainly linked to aggressive tumor behavior, immune evasion, and therapy resistance. In contrast, FOXP2 and FOXP1 appear to suppress tumor growth. However, evidence for their functions is limited. The tumor-suppressive mechanisms of these factors in thyroid cancer remain incompletely delineated. The dual, tissue-specific roles of FOXP family members point to their importance as biomarkers and treatment targets in thyroid cancer.

To the best of our knowledge, this review represents one of the first attempts to comprehensively synthesize the roles of all FOXP family members in thyroid cancer. Despite accumulating evidence, several research gaps still exist. Future studies should use large, well-characterized patient cohorts to validate FOXP’s prognostic and predictive value. Mechanistic investigations must clarify FOXP-driven regulatory networks, including interactions with oncogenic pathways and the tumor microenvironment. FOXP-targeted or FOXP-modulating therapies, such as RNA-based approaches and pathway-specific inhibitors, are promising. Incorporating FOXP expression into multimodal risk stratification may improve individualized treatment. An expanded understanding of FOXP biology may offer new ways to advance the diagnosis, prognosis, and treatment of thyroid cancer. Ultimately, integrating FOXP-centered molecular knowledge of clinical decision-making may constitute an important step toward precision oncology in thyroid cancer.

## Figures and Tables

**Table 1:** FOXP1 protein isoforms.

**FOXP1**	**Isoform**	**Length (nt)**	**Length (aa)**	**Conserved Domain**	**Location of Conserved Domain**
	A	7177 6808 7004 7082 7088	677	Transcription factor of the Forkhead/HNF3 family	437 → 651
				FOXP coiled-coil domain	302 →369
				Forkhead (FH) domain	464 → 545
	B	882	114	/	/
	C	7174	676	Transcription factor of the Forkhead/HNF3 family	437 → 650
				FOXP coiled-coil domain	302 → 369
				Forkhead (FH) domain	463 → 544
	D	7136	693	FOXP coiled-coil domain	302 → 369
				Forkhead (FH) domain	464 → 545
	E	6510	601	Transcription factor of the Forkhead/HNF3 family	361 → 575
				FOXP coiled-coil domain	226 → 293
				Forkhead (FH) domain	388 → 469
	F	6392 6482 6500	577	Transcription factor of the Forkhead/HNF3 family	337 → 551
				FOXP coiled-coil domain	202 → 269
				Forkhead (FH) domain	364 → 445
	I	6479 6497 6389	576	Transcription factor of the Forkhead/HNF3 family	336 → 550
				FOXP coiled-coil domain	201 →268
				Forkhead (FH) domain	363 → 444
	J	7174	676	Transcription factor of the Forkhead/HNF3 family	436 → 650
				FOXP coiled-coil domain	301 → 368
				Forkhead (FH) domain	463 → 544
	K	6479	576	Transcription factor of the Forkhead/HNF3 family	337 → 550
				FOXP coiled-coil domain	202 → 269
				Forkhead (FH) domain	363 → 444

nt—nucleotide; aa—amino acid.

**Table 2:** FOXP2 protein isoforms.

**FOXP2**	**Isoform**	**Length (nt)**	**Length (aa)**	**Conserved Domain**	**Location of Conserved Domain**
	I	6543	715	FOXP coiled-coil domain	342 → 409
				Transcription factor of the Forkhead/HNF3 family	477 → 708
	II	6618	740	FOXP coiled-coil domain	367→ 434
				Transcription factor of the Forkhead/HNF3 family	502 → 733
	III	1410	432	FOXP coiled-coil domain	342 → 409
	IV	6594	732	FOXP coiled-coil domain	359 → 426
				Transcription factor of the Forkhead/HNF3 family	494 → 725
	V	6540	714	FOXP coiled-coil domain	341 → 408
				Transcription factor of the Forkhead/HNF3 family	476 → 707
	VI	1485	457	FOXP coiled-coil domain	367 → 434

nt—nucleotide; aa—amino acid.

**Table 3:** FOXP4 protein isoforms.

**FOXP4**	**Isoform**	**Length (nt)**	**Length (aa)**	**Conserved Domain**	**Location of Conserved Domain**
	1	5994	680	FOXP coiled-coil domain	303 → 370
				Transcription factor of the Forkhead/HNF3 family	364 → 560
	2	5955	667	FOXP coiled-coil domain	302 → 369
				Forkhead (FH) domain	454 → 540
	3	5988	678	FOXP coiled-coil domain	301 → 368
				Transcription factor of the Forkhead/HNF3 family	362 → 558
	4	5958	668	FOXP coiled-coil domain	303 → 370
				Forkhead (FH) domain	455 → 541
	5	5898	648	FOXP coiled-coil domain	271 → 338
				Transcription factor of the Forkhead/HNF3 family	332 → 528
	6	5862	636	FOXP coiled-coil domain	271 → 338
				Forkhead (FH) domain	423 → 509
	X1	6015 7915 7928	687	FOXP coiled-coil domain	310 → 377
				Transcription factor of the Forkhead/HNF3 family	371 → 567
	X2	6009	685	FOXP coiled-coil domain	308 → 375
				Transcription factor of the Forkhead/HNF3 family	369 → 565
	X3	5991	679	FOXP coiled-coil domain	302 → 369
				Transcription factor of the Forkhead/HNF3 family	363 → 559
	X4	5979	675	FOXP coiled-coil domain	310 → 377
				Forkhead (FH) domain	462 → 548
	X5	5952	666	FOXP coiled-coil domain	301 → 368
				Forkhead (FH) domain	453 → 539
	X6	5919	655	FOXP coiled-coil domain	278 → 345
				Transcription factor of the Forkhead/HNF3 family	339 → 535
	X7	5330 5379	581	FOXP coiled-coil domain	204 → 271
				Transcription factor of the Forkhead/HNF3 family	265 → 461
	X8	5319	562	FOXP coiled-coil domain	197 → 264
				Forkhead (FH) domain	349 → 435

nt—nucleotide; aa—amino acid.

**Table 4:** Summary of bioinformatic and TCGA-derived findings regarding FOXP family members in thyroid cancer.

FOXP Member	Dataset/Study	Main Molecular Findings	Reported Clinical Associations	References
FOXP3	Clinical cohort and expression-based studies in PTC/DTC	Increased FOXP3 expression in tumor cells and tumor-infiltrating lymphocytes; altered nuclear and cytoplasmic localization	Lymph node metastasis, extrathyroidal extension, aggressive phenotype, shorter DFS/OS, immune evasion, radioiodine resistance	[101,102,103,104,105]
FOXP4	TCGA-THCA dataset and experimental validation studies (Zhou et al.)	Increased FOXP4 mRNA and protein expression in thyroid cancer tissues; association with Wnt/β-catenin, Akt/mTOR, and EMT-related signaling	Vascular invasion, extrathyroidal invasion, distant metastasis, poor prognosis	[106,107]
FOXP2	Transcriptomic, network-based, and single-cell RNA sequencing analyses	Context-dependent dysregulation of FOXP2; involvement in apoptosis, stromal signaling, Hedgehog and Wnt/β-catenin pathways	Altered proliferation, apoptosis, immune-related interactions, tumor microenvironment modulation	[108,109,110,111,112,113]
FOXP1	DTC clinical expression study	Increased FOXP1 mRNA expression with reduced protein levels, suggesting post-transcriptional regulation	Potential tumor suppressor role in thyroid cancer	[114]

## Data Availability

New data were created or analyzed in this study.

## Abbreviations

The following abbreviations are used in this manuscript:

PCLBCL	Primary Cutaneous Large B-cell Lymphoma
TI-RADS	Thyroid Imaging Reporting and Data System
ceRNA	Competing Endogenous RNA
CtBP1	C-terminal Binding site for C-terminal binding protein 1
DHGTC	Differentiated High-grade Thyroid Carcinoma
DLBCL	Diffuse Large B-cell Lymphoma
FBXW7	F-box and WD repeat domain containing 7
iTregs	Induced Regulatory T cells
nTregs	NaturalRegulatory T cells
TGF-β1	Transforming Growth Factor-β1
AML-1	Acute Myeloid Leukemia 1
IGF-1	Insulin-like Growth Factor 1
IL-10	Interleukin-10
NF-κB	Nuclear factor kappa-light-chain-enhancer of activated B cells
MALT	Gastric Mucosa-Associated Lymphoid Tissue Lymphoma
PDTC	Poorly Differentiated Thyroid Carcinoma
Treg	Regulatory T cells
AML	Acute Myeloid Leukemia
ATC	Anaplastic Thyroid Carcinoma
CLT	Chronic Lymphocytic Thyroiditis
DTC	Differentiated Thyroid Carcinomas
EMT	Epithelial–Mesenchymal Transition
FTC	Follicular Thyroid Carcinoma
HCC	Hepatocellular Carcinoma
MDA	Mucinous Minimal Deviation Adenocarcinoma
MTC	Medullary Thyroid Carcinoma
NIS	Sodium Iodide Symporter
OTC	OncocyticThyroidCarcinoma
PTC	Papillary Thyroid Carcinoma
SNPs	Single Nucleotide Polymorphisms
TKI	Tyrosine Kinase Inhibitors
WHD	Winged Helix Domain
WHO	World Health Organization
HT	Hashimoto’s Thyroiditis
LT	Lymphocytic Thyroiditis
TC	Thyroid Cancer
